# Characterisation and Evaluation of Trimesic Acid Derivatives as Disulphide Cross-Linked Polymers for Potential Colon Targeted Drug Delivery

**DOI:** 10.3390/polym9080311

**Published:** 2017-07-27

**Authors:** Siti Nur Aishah Mat Yusuf, Yoke Mooi Ng, Asila Dinie Ayub, Siti Hawa Ngalim, Vuanghao Lim

**Affiliations:** 1 Integrative Medicine Cluster, Advanced Medical and Dental Institute, Universiti Sains Malaysia, Bertam, 13200 Kepala Batas, Penang, Malaysia; nuraishahyusuf@gmail.com (S.N.A.M.Y.); yumei.wu91@gmail.com (Y.M.N.); asiladinie@gmail.com (A.D.A.); 2 Regenerative Medicine Cluster, Advanced Medical and Dental Institute, Universiti Sains Malaysia, Bertam, 13200 Kepala Batas, Penang, Malaysia; siti.hawa.ngalim@usm.my; 3 Faculty of Engineering Technology, Universiti Malaysia Perlis, UniCITI Alam Campus, 02100 Padang Besar, Perlis, Malaysia

**Keywords:** trimesic acid, disulphide cross-linked polymer, reduction response, colon targeted delivery

## Abstract

Discovery and use of biocompatible polymers offers great promise in the pharmaceutical field, particularly in drug delivery systems. Disulphide bonds, which commonly occur in peptides and proteins and have been used as drug-glutathione conjugates, are reductively cleaved in the colon. The intrinsic stability of a disulphide relative to thiol groups is determined by the redox potential of the environment. The objective of this study was to synthesise a trimesic acid-based disulphide cross-linked polymer that could potentially be used for targeted delivery to the colon. The monomer was synthesised by an amide coupling reaction between trimesic acid and (triphenylmethyl) thioethylamine using a two-step synthesis method. The *s*-trityl group was removed using a cocktail of trifluoroacetic acid and triethylsilane to expose the thiols in preparation for further polymerisation. The resulting polymers (P10, P15, P21, P25, and P51, generated using different molar ratios) were reduced after 1.5 h of reduction time. Scanning electron microscopy images of the polymers showed spherical, loose, or tight patterns depending on the molar ratio of polymerisation. These polymers also exhibited efficient dissolution under various gastrointestinal conditions. Of the five polymers tested, P10 and P15 appeared to be promising drug delivery vehicles for poorly soluble drugs, due to the hydrophobic nature of the polymers.

## 1. Introduction

The increasing demand for site-specific delivery systems has been fuelled by the discovery of peptide and protein drugs [1]. Developing medications and frameworks for their conveyance to the colon of the gastrointestinal tract (GIT) has become a prominent research goal in recent years. Targeting drugs to the colon is useful for the delivery of low molecular weight compounds such as chemotherapeutic drugs [2]. Delivery of drugs for systemic dissemination through colonic ingestion represents a novel method of administering peptide and protein drug molecules and medications that are ineffectively passed along from the upper GIT. 

Coating drugs with polymers is a simple approach for colon-specific drug delivery. Examples include biodegradable polymers, pH dependent polymers, and delivery systems based on the metabolic activity of colonic bacteria. The latter two in particular have garnered scientific interest [3], as azo, polysaccharide, and disulphide polymers degrade either in the presence of bacterial enzymes or in the low redox potential in the colon [4].

Azo polymers were the first coating materials developed with the ability to biodegrade in the colon. However, delayed absorption of the proteins due to slow disintegration of the coating in the colon by bacterial enzymes was reported [5]. Polymers with a disulphide backbone function differently; they are degraded into smaller thiols after exposure to the low redox potential in the colon. Further hydrolysis of these linkages yields the parent amino acid, which reduces potential toxicity that may result from polymer remnants [4].

Studies of disulphide polymers have improved the outcome of drug delivery. Use of such polymers reduces toxicity and side effects of the carrier, can lower the dosage while improving the bioavailability [6], and may provide better circulation time and in vivo targeting [7]. Previous studies described disulphide nanocarriers capable of encapsulating a drug [8] and releasing a drug under reduced conditions [9]. Also the carriers were stable in the presence of various pH levels, electrolytes, and common enzymes [10].

The goal of this study was to test the effectiveness of trimesic acid derivative disulphide-containing polymers for colon-targeted drug delivery. Trimesic acid is mainly used as an adhesive and in coating materials. It is also used as pharmaceutical intermediates, such as drugs and gene carriers. Several types of trimesic acid-based dendrimers have been described as in vivo biomolecule delivery agents [11]. Trimesic acid was chosen for this study because it can crystallise from water in a hydrogen-bonded hydrated network. Therefore, the synthesised polymers will provide the desired solubility after the polymerisation process.

## 2. Materials and Methods

### 2.1. Materials

Triphenylmethanol, cysteamine dihydrochloride, trifluoroacetic acid (TFA), trimesic acid, hydroxybenzotriazole (HOBT), 1-ethyl-3-(3-dimethylaminopropyl)carbodiimide (EDAC), *N*,*N*-Diisopropylethylamine (DIPEA), triethylsilane (TES), 2,2′-(ethylenedioxy)diethanethiol, dithiothreitol, ninhydrin, Ellman’s reagent, pepsin, pancreatin, deuterated chloroform, dimethyl sulfoxide (DMSO) and deuterated dimethyl sulfoxide (DMSO-d_6_) were purchased from Sigma (Hamburg, Germany). Diethyl ether, dichloromethane (DCM), petroleum ether, ethyl acetate, acetic acid, and hydrochloric acid were purchased from QRec Chemical (Chonburi, Thailand). Sodium chloride, sodium bicarbonate, and sodium hydroxide were obtained from R&M Chemical, Essex, United Kingdom. Dimethyl formamide was obtained from Fisher Scientific (Leicestershire, UK). Wilkens chalgren anaerobic broth was obtained from Oxoid (Hampshire, UK).

### 2.2. Synthesis of (Triphenylmethyl)thioethylamine *(**1**)*

(Triphenylmethyl)thioethylamine was synthesised by stirring 0.05 mol of cysteamine dihydrochloride with 0.05 mol of triphenylmethanol in 50 mL of TFA. The reaction was performed at room temperature for 3 h with protection from moisture. The reaction mixture was then evaporated using an Eyela N-1200BV-WD rotary evaporator (Tokyo, Japan) at 65 °C until the mixture became thick and brownish in colour. The mixture then was washed with diethyl ether until a white solid appeared and no brown colour could be seen. The white precipitate was filtered and left to dry overnight. The dried white solid was dissolved in diethyl ether and washed again with 1 M sodium hydroxide. The ether phase was collected and concentrated using a rotary evaporator [12]. The white solid obtained was used to synthesise compound **2**. 

### 2.3. Synthesis of N^1^,N^3^,N^5^-tris(2-(Tritylthio)ethyl)benzene-1,3,5-tricarboxamide *(**2**)*

First, 0.006 mol of trimesic acid was dissolved in dimethylformamide. The trimesic acid solution was mixed and stirred with 0.018 mol of (triphenylmethyl)thioethylamine in DCM at 0 °C for 48 h in an inert atmosphere. The reaction was catalysed by 0.018 mol of DIPEA, 0.018 mol of HOBT, and 0.018 mol of EDAC. The yield was filtered, washed with 5% citric acid, concentrated sodium bicarbonate, and concentrated sodium chloride, and evaporated using the EZ-2 Elite personal evaporator (Genevac, Suffolk, UK). The compound of interest was purified by column chromatography, using petroleum ether:ethyl acetate, ratio 65:35 as the mobile phase. The purified compound was then subjected to removal of the *S*-trityl protecting group. 

### 2.4. Synthesis of N^1^,N^3^,N^5^-tris(2-Mercaptoethyl)benzene-1,3,5-tricarboxamide *(**3**)*

Removal of the *s*-trityl protecting group was carried out by stirring the purified compound with TFA and triethylsilane cocktail in DCM for 6 h. The reaction mixture was evaporated using the rotary evaporator, and the product was washed with copious amounts of petroleum ether.

### 2.5. Characterisation of the Monomer

#### 2.5.1. Fourier-Transform Infra-Red Spectroscopy

Fourier-transform infra-red spectroscopy (FTIR) spectra using a KBr disc were obtained using a Nexus 670 Thermo Nicolet (Madison, WI, USA) spectrometer in the 500–4000 cm^−1^ spectral region. Samples were finely ground before performing the analysis.

#### 2.5.2. Nuclear Magnetic Resonance 

Both ^13^C nuclear magnetic resonance (NMR) and ^1^H NMR were measured using a Bruker Avance 500 (Stuttgart, Germany) spectrometer operated at 125 and 500 MHz, respectively. Deuterated chloroform was used as the solvent for compounds **1** and **2**, whereas deuterated DMSO was used for compound **3**.

#### 2.5.3. Elemental Analysis

The elemental analysis was conducted by combustion reaction in a CHNS/O analyser (Perkin-Elmer 2400, Madison, WI, USA) with a combustion temperature of 950 °C and reduction at 550 °C. 

#### 2.5.4. Melting Point Analysis

Melting points of compounds **1**, **2**, and **3** were determined using a Stuart MP10 melting point analyser (Staffordshire, UK). Samples were inserted into the melting point capillary and observed under increasing temperature. 

### 2.6. Oxidation of the Monomer to Form Polymers

Compound **3** was stirred in ammonium bicarbonate buffer (0.1 M, pH 8.3). DMSO was slowly added until approximately 50% of the solids were dissolved. The solution was stirred continuously in open air. The reaction was terminated when no purple colour was observed after testing with 5% sodium nitroprusside. The final suspension was filtered and washed with distilled water and methanol to produce a solid powder. Different molar ratios were used for oxidation of **3** with 2,2′-(ethylenedioxy)diethanethiol (dithiol) as follows;

P10: **3** only
P15: 1 mol of **3**: 5 mol of dithiol
P21: 2 mol of **3**: 1 mol of dithiol
P25: 2 mol of **3**: 5 mol of dithiol
P51: 5 mol of **3**: 1 mol of dithiol

### 2.7. Physical Characterisation of the Polymer

#### 2.7.1. Raman Spectrometry

Raman spectra were recorded using a Jobin Yvon HR 800 UV Raman spectrometer (Lower Hutt, New Zealand). The incident laser excitation wavelength was 514.5 nm with an output of 20 mW. The samples were placed in a circle form on a glass slide, and spectra were recorded from 100 to 3000 cm^−1^ for identification of S–H and S–S peaks.

#### 2.7.2. Scanning Electron Microscopy-Energy Dispersive X-ray 

In scanning electron microscopy (SEM) analysis, the surface of the sample was coated with chromium using a sputter coater (Quorum, Oxford, UK). Images up to 12,000× magnification were taken using a SEM microscope (Quantafeg 650, Oxford, UK). Energy dispersive X-ray (EDX) was performed using the detection microanalysis system (Oxford Instruments PLC, Bucks, UK). 

### 2.8. Chemical Reduction Studies

A total of 0.1 g of polymer and 1.3 mL of 34.3 mM acetic acid were dissolved in 10 mL of distilled water. The mixture in a three-neck round bottom flask was refluxed to 100 °C in an oil bath, and 1.95 g of 30 mM zinc dust was added slowly while stirring. Next, 10 µL of the sample was withdrawn from the side arm using a high performance liquid chromatography (HPLC) microsyringe and diluted in distilled water at a dilution factor of 2.5 × 10^3^. The diluted sample was vortexed and filtered through cotton wool. The filtrate was then used for the measurement of thiol concentration using Ellman’s reagent.

### 2.9. Measurement of Thiol Concentration

Ellman’s reagents was prepared by dissolving 4 mg of Ellman’s reagent in 1 mL of SØrensen’s phosphate buffer (pH 7.4). A sample tube containing 2.5 mL of SØrensen’s phosphate buffer and 50 µL of Ellman’s reagent was prepared. Next, 250 µL of sample was added into each sample tube; 250 µL of SØrensen’s phosphate buffer was added for the blank measurement. The tubes were vortexed and left at room temperature for 15 min to allow the thiol exchange to occur. UV absorbance was measured at 412 nm using a 1 cm cuvette (Perkin-Elmer Lambda 25 UV/VIS Spectrometer, Madison, WI, USA). The concentration of thiol can be determined by calculation using the Beer-Lambert equation [13]:

C = A/ε·d

where C is concentration (M), A is absorbance, d is cell path length (1 cm), and ε is the molar absorption coefficient in SØrensen’s phosphate buffer pH 7.4 (14, 150 M^−1^·cm^−1^).

### 2.10. In Vitro Dissolution Studies

All simulated condition experiments were conducted in triplicate. All samples were subjected to measurement of thiol concentration.

#### 2.10.1. Preparation of *Bacteroides fragilis* (*B. fragilis*) Culture

A single colony from plate culture was sub-cultured in 100 mL of Wilkens-Chalgren anaerobic broth. The cultured flask was placed in anaerobic chamber and culture for 24 h at 37 °C without agitation.

#### 2.10.2. Incubation of Polymers in the Simulated Gastric Condition

The gastric condition was simulated by preparing a solution containing 30 mL of 0.1 M hydrochloric acid pH 1.0 and 0.32% (*w/v*) pepsin. The fluid was incubated in a water bath at 37 °C. A visking dialysis tube containing 0.3 g of polymers was inserted into the flask for 2 h. Samples were taken every 30 min. For every 250 µL sample taken, 250 µL of simulated gastric fluid was added to the reaction flask. 

#### 2.10.3. Incubation of Polymers in the Simulated Intestinal Condition

The dialysis tube from the simulated gastric condition was transferred into a new pre-warmed (37 °C) conical flask containing 30 mL of phosphate buffer pH 7.4 and 1% (*w/v*) pancreatin to simulate the intestine condition. The intestinal incubation lasted for 3 h. Samples were withdrawn every 30 min. For every 250 µL sample taken, 250 µL of simulated intestinal fluid was added to the reaction flask.

#### 2.10.4. Incubation of Polymers in the Simulated Colon Condition

For the simulated colon condition, the dialysis tube was opened and a bacterial pellet from a 100 mL *B. fragilis* culture was inserted. The dialysis sac containing the polymer sample and the bacterial pellet was then transferred into a pre-flushed oxygen-free nitrogen flask containing 30 mL of phosphate buffer pH 6, which mimicked the colon condition. The incubation was continued in a shaking water bath at 37 °C with continuous flow of oxygen-free nitrogen. Samples were taken at 1, 2, 3, 4, 6, 9, 13, 17, 21 and 25 h. For every 250 µL sample taken, 250 µL of simulated colon fluid was added to the reaction flask.

#### 2.10.5. Control Incubation

Two control experiments for the simulated colon condition were conducted. The first used only the polymer, without bacterial incubation. The second involved incubation of the bacterial pellet only. They were conducted separately in buffer pH 6. 

### 2.11. Statistical Analysis

Statistical analyses were performed using IBM SPSS statistics 22.0 (SPSS Inc., Chicago, IL, USA). Final thiol concentration in every simulated condition and various sets of simulated colon conditions were analysed using one-way analysis of variance. Post-hoc analysis was conducted using Dunnett’s *t*-test (2-sided) and Dunnett’s T3 test when statistically significant (*p* < 0.05) results were obtained. 

## 3. Results

### 3.1. Synthesis and Physical Characterisation of the Thiolated Monomer

**Figure 1:**
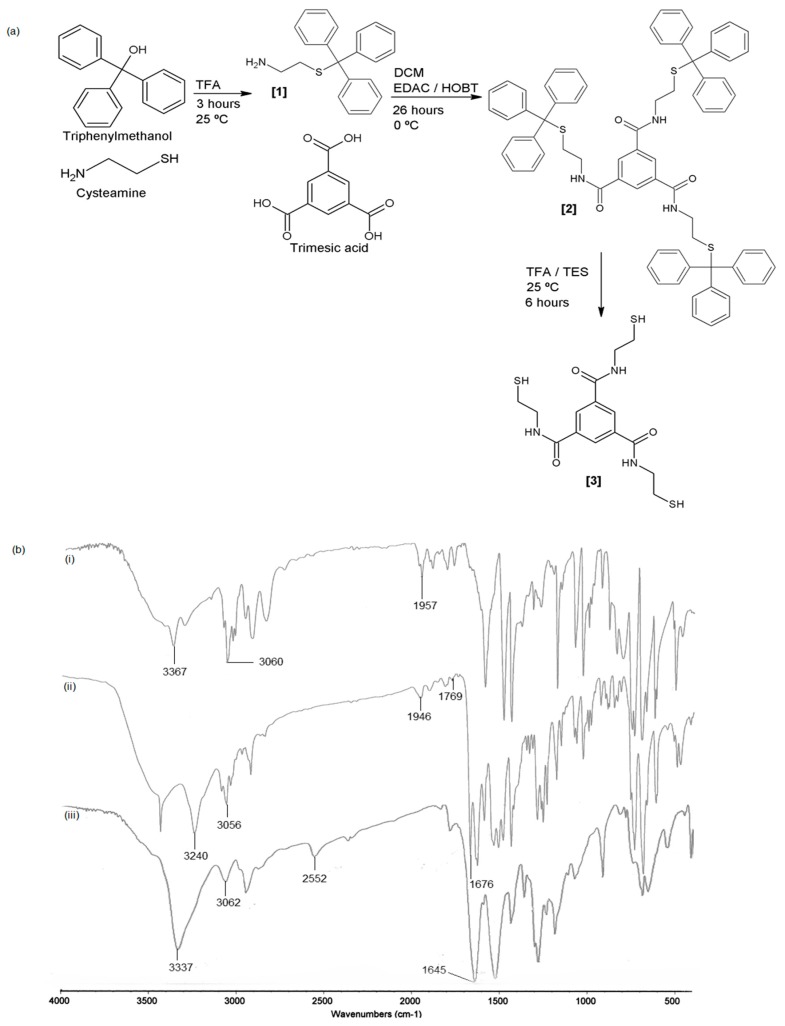
(**a**) Synthetic routes for preparation of the thiolated monomer; (**b**) FTIR spectra of (**i**) (triphenylmethyl)thioethylamine, (**ii**) *N*^1^*,N*^3^*,N*^5^-tris(2-(Tritylthio)ethyl)benzene-1,3,5-tricarboxamide, and (**iii**) *N*^1^*,N*^3^*,N*^5^-tris(2-Mercaptoethyl)benzene-1,3,5-tricarboxamide

Figure 1a illustrates the synthesis of thiolated monomer. Compound **1** was synthesised from triphenylmethanol and cysteamine with percentage yields of 92–95%. Thin layer chromatography of compound **1** showed a spot with R_f_ 0.7, and the ninhydrin test was positive, which indicated the presence of primary amine [14]. Compound **2**, which was synthesised from compound **1** and trimesic acid via amide linkage, had a medium yield (56–60%). The remaining trimesic acid and bi-product were removed by partitioning with 5% citric acid, concentrated sodium bicarbonate, and concentrated sodium chloride. Isolation of the compound of interest (**3**) was completed via column chromatography using petroleum ether and ethyl acetate as the mobile phase. The removal of the *s*-trityl group from **2** produced **3** with yield of 85–90%. All three compounds were in the form of a white powder. The three synthesised compounds (**1**,**2**,**3**) were confirmed with FTIR, NMR, and elemental analysis. The spectra show peaks at 1957, 3060, and 3367 cm^−1^, which correspond to benzene, CH stretching, and amine stretching, respectively (Figure 1b(i)). Compound **2** was successfully synthesised, as indicated by the presence of a peak at 1676 cm^−1^ that shows the amide bonding between the carboxyl group of trimesic acid and amine from **1**. Removal of the *s*-trityl group from **2** exposed the free thiol, which is shown by a peak at 2552 cm^−1^ (Figure 1b(iii)).

**Figure 2:**
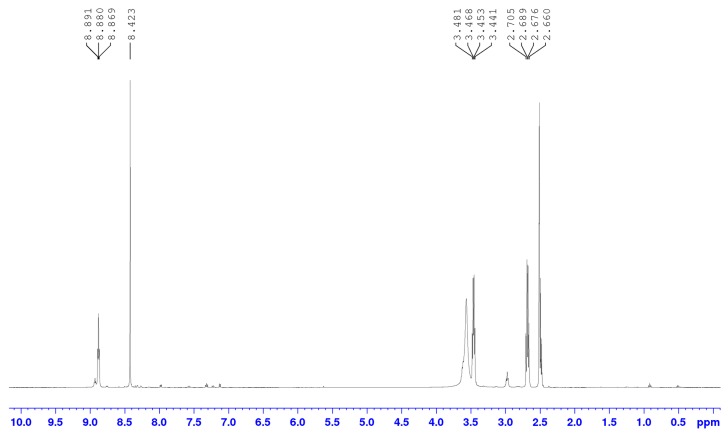
^1^H NMR spectra of *N*^1^*,N*^3^*,N*^5^-tris(2-Mercaptoethyl)benzene-1,3,5-tricarboxamide (**3**) in DMSO-d_6_.

The ^1^H NMR spectra of compound **1** in CDCl_3_, δ 2.339–2.365 (2H, t, CH_2_N, *J* = 6.5 Hz), 2.612–2.639 (2H, t, CH_2_S, *J* = 6.5 Hz), 7.224–7.471 (m, 15H aromatic) shows that **1** was successfully synthesised. The appearance of a proton peak at δ 6.273–6.296 (3H, t, CONH, *J* = 6.0 Hz) and 7.107–7.355 (m, 45H aromatic C–H) shows that **1** and trimesic acid are successfully linked. Figure 2 shows the ^1^H NMR spectrum of **3** in DMSO-d_6_, δ 2.660–2.705 (2H, q, CH_2_N, *J* = 7.5 Hz), 3.441–3.481 (2H, q, CH_2_S, *J* = 6.5 Hz), 8.423 (s, CH), 8.869–8.891 (3H, t, CONH, *J* = 5.5 Hz).

Elemental analysis was performed to determine the weight percentage of an element in a compound. CHN analysis is very useful for organic compounds containing C–C bonds [8]. Table 1 shows the weight percentage of carbon, hydrogen, and nitrogen and the melting point of all synthesised compounds. For CHN analysis, the obtained experimental percentages were similar to the theoretical percentages which were calculated from the empirical formulas of the synthesised compounds. These results indicate the efficient synthesis of compunds (**1**), (**2**) and (**3**) because they are in close agreements with FTIR and NMR results. A narrow melting point of all compounds indicates their purity in the solid state. 

### 3.2. Characterisation of the Disulphide Polymers

#### 3.2.1. Physical Characterisation and Solubility

Polymerisation of the monomer was carried out via air oxidation [15] using DMSO as the oxidising agent [10]. The reaction was terminated when a negative result was obtained from sodium nitroprusside, which indicated successful formation of disulphide bonds. Raman spectrometry is an important method to confirm the formation of the disulphide linkage. The Raman spectra of compound **3** and its polymers were recorded in the range of 100 to 3000 cm^−1^. Major peaks identified included an S–H peak at 2550 cm^−1^ (compound **3**) and an S–S peak at 500 to 510 cm^−1^ (polymers). The results confirmed the successful formation of disulphide bonds in the polymers. Physical appearance and solubility of the synthesised disulphide polymers are described in Table 2 and Table 3.

#### 3.2.2. Fourier-Transform Infrared Spectroscopy

All polymers were characterised by FTIR. The results showed the appearance of the C–O–C stretch peak of the dithiol monomer (2,2′-(ethylenedioxy)diethanethiol) for polymer P15 (1102 cm^−1^), P21 (1108 cm^−1^), P25 (1109 cm^−1^), and P51 (1109 cm^−1^). These results confirmed that oxidation between trithiol and dithiol monomers occurred successfully. The C–O–C stretch was not observed in P10, as P10 polymerisation involves only the trithiol monomer. The FTIR results for all disulphide cross-linked polymers are summarised as follows: FTIR (KBr disc); P10 = 3415 cm^−1^ (–NH stretching), 3075 cm^−1^ (–CH_2_), 1750 cm^−1^ (aromatic C), 1648 cm^−1^ (–NHCO), 1437 cm^−1^ (–CN), P15 = 3337 cm^−1^ (–NH stretching), 3060 cm^−1^ (–CH_2_), 1835 cm^−1^ (aromatic C), 1644 cm^−1^ (–NHCO), 1436 cm^−1^ (–CN), 1102 cm^−1^ (C–O–C stretch); P21 = 3337 cm^−1^ (–NH stretching), 3061 cm^−1^ (–CH_2_), 1850 cm^−1^ (aromatic C), 1644 cm^−1^ (–NHCO), 1436 cm^−1^ (–CN), 1108 cm^−1^ (C–O–C stretch); P25 = 3342 cm^−1^ (–NH stretching), 3057 cm^−1^ (–CH_2_), 1758 cm^−1^ (aromatic C), 1644 cm^−1^ (–NHCO), 1446 cm^−1^ (–CN), 1109 cm^−1^ (C–O–C stretch), and P51 = 3333 cm^−1^ (–NH stretching), 3060 cm^−1^ (–CH_2_), 1850 cm^−1^ (aromatic C), 1648 cm^−1^ (–NHCO), 1436 cm^−1^ (–CN), 1109 cm^−1^ (C–O–C stretch).

#### 3.2.3. SEM-EDX Micrographs

**Figure 3:**
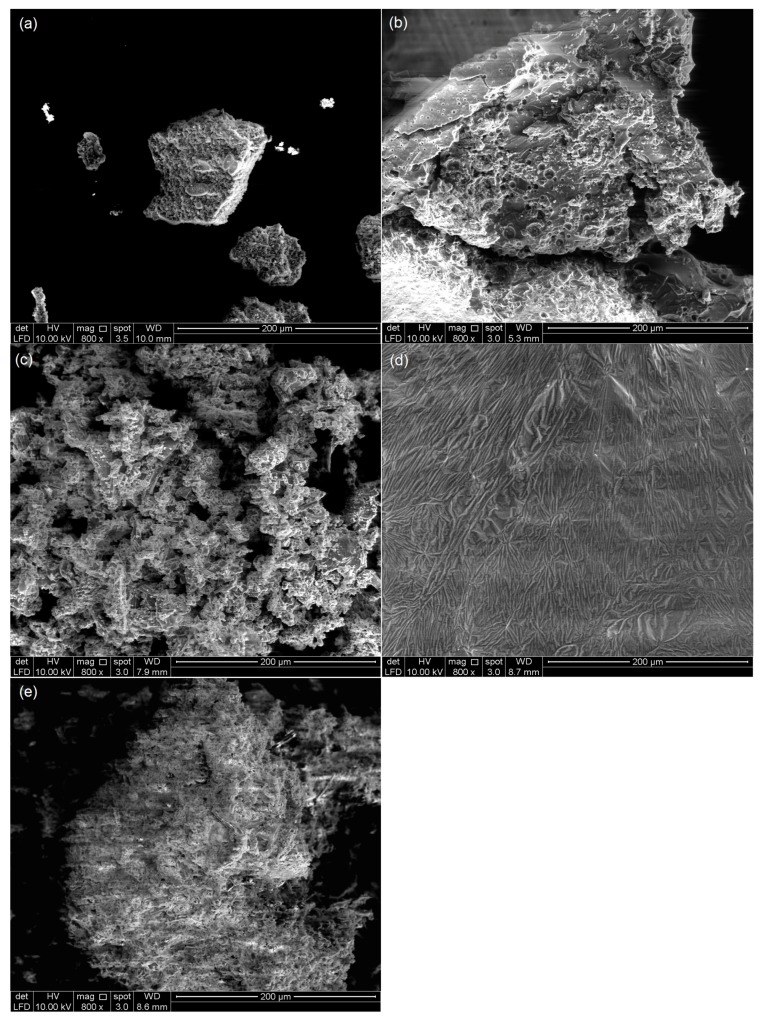
Scanning Electron microscopy (SEM) images at 800x magnification of polymer (**a**) P10, (**b**) P15, (**c**) P21, (**d**) P25, and (**e**) P51.

**Figure 4:**
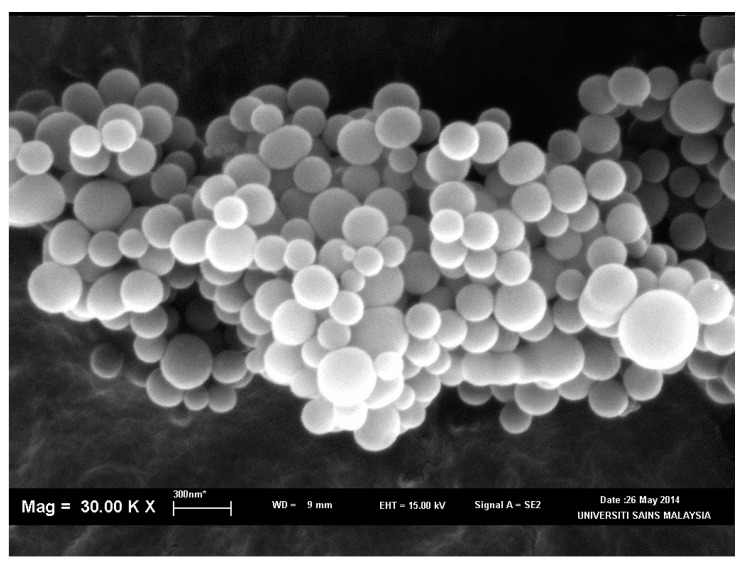
SEM image of polymer P10 at 30,000× magnification.

**Figure 5:**
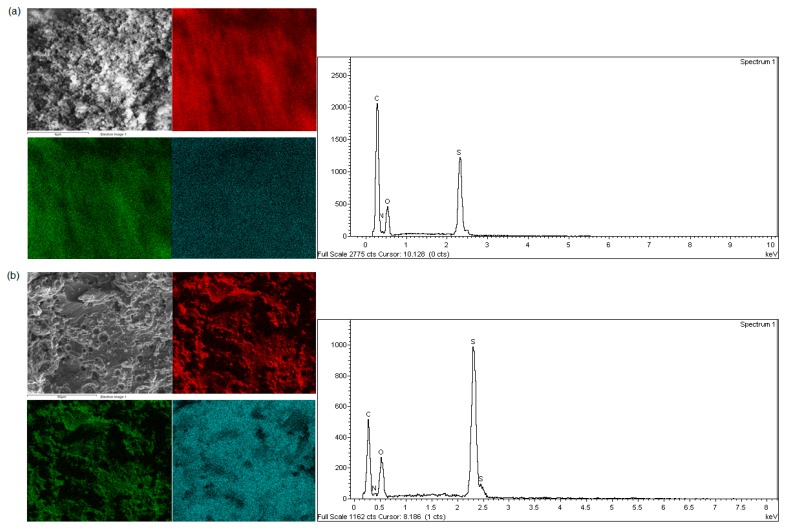

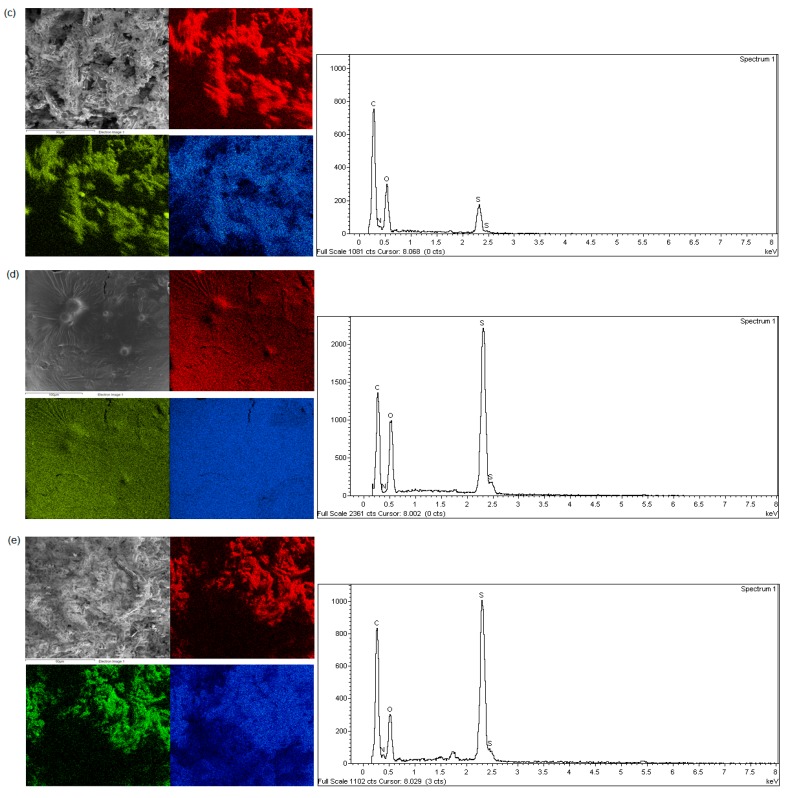
SEM micrographs, EDX, and elemental mapping for carbon (red), oxygen (green), and sulphur (blue) of polymer (**a**) P10, (**b**) P15, (**c**) P21, (**d**) P25, and (**e**) P51.

The surface morphology of all polymers was investigated by analysing SEM micrographs. EDX and mapping were used to determine the distribution of the elements [16]. Figure 3 shows images of all polymers at magnification of 800x. Polymers with a higher molar ratio of trithiol had a rough and coarse surface (i.e., P21 (c) and P51 (e)) [12,17]. P51 exhibited a tighter polymer surface compared to P21, due to a greater trithiol ratio during oxidative polymerisation. The polymerisation between trithiol and dithiol yielded a loose polymer, as seen in P15 (b) and P25 (d). P15 had a more porous surface compared to all other polymers, due to its high molar ratio of dithiol [12,17,18]. P10 exhibited a different morphology, as it formed a nanoparticle with size range of 65 nm to 1 µm (Figure 4). The formation of nanoparticle following trithiol self-polymerisation was not observed in previous studies [4,12,18,19]. This result was unique and novel to our finding. These SEM results agreed with previous studies reporting that tight polymers have a rough surface [12,17,19]. EDX microscopy illustrated the distribution of carbon, oxygen, and sulphur, which was supported by elemental mapping (Figure 5). Carbon, oxygen, and sulphur were uniformly distributed in all synthesised polymers.

#### 3.2.4. Chemical Reduction of Disulphide Cross-Linked Polymers

**Figure 6:**
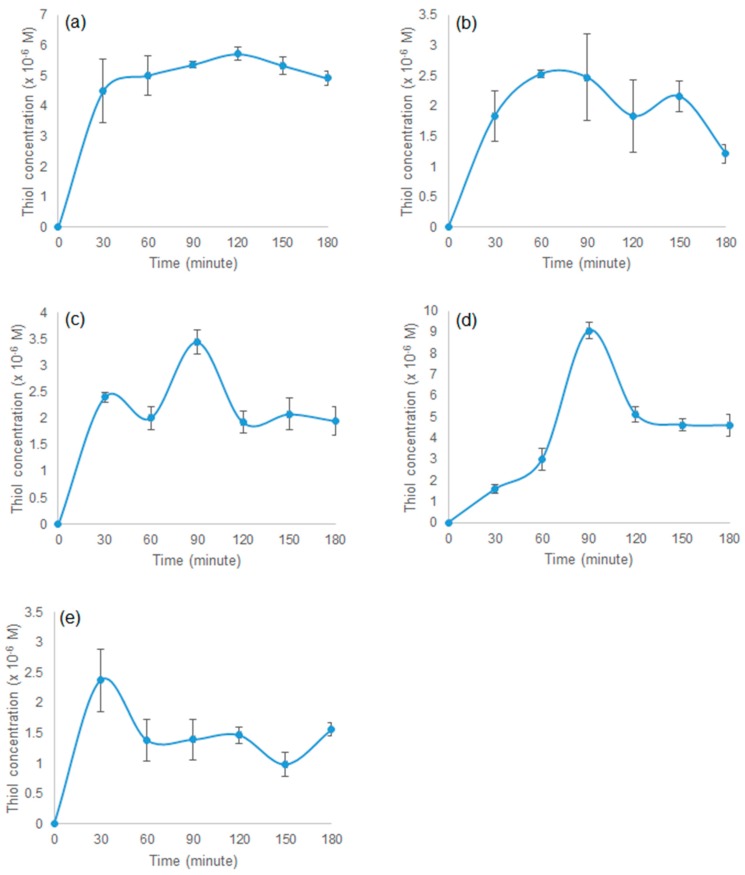
Chemical reduction of polymer (**a**) P10, (**b**) P15, (**c**) P21, (**d**) P25, and (**e**) P51, mean ± SEM, n = 3.

Chemical reduction of all polymers was carried out for three hours, and the results are shown in Figure 6. The highest thiol concentration was found in polymer P10 and the lowest was seen in polymer P15. The average thiol concentration of polymer P10 was approximately 4.923 × 10^−6^ M, followed by P25 (≈4.226 × 10^−6^ M), P21 (≈1.953 × 10^−6^ M), P51 (≈1.562 × 10^−6^ M), and P15 (≈ 1.215 × 10^−6^ M). Polymer P10 showed stable reduction compared to the other polymers. This result differed from those of previous studies reporting that the maximum thiol concentration was observed in polymer P15 [12,19]. The larger surface area of polymer P10 contributed to its higher thiol concentration. Generally, the maximum thiol concentration was achieved after 90 min of reduction and reached a plateau at three hours. The reduction studies showed that all synthesised polymers were capable of being reduced and releasing free thiol.

#### 3.2.5. In Vitro Dissolution Studies

**Figure 7:**
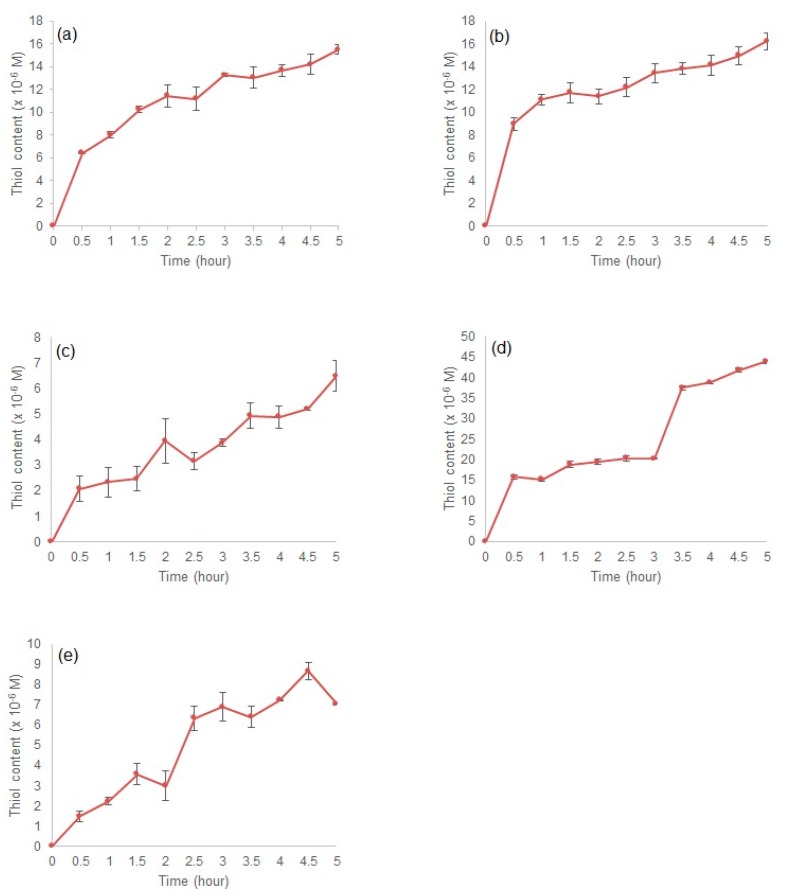
Thiol concentration in simulated gastric and intestinal conditions for polymer (**a**) P10, (**b**) P15, (**c**) P21, (**d**) P25, and (**e**) P51.

**Figure 8:**
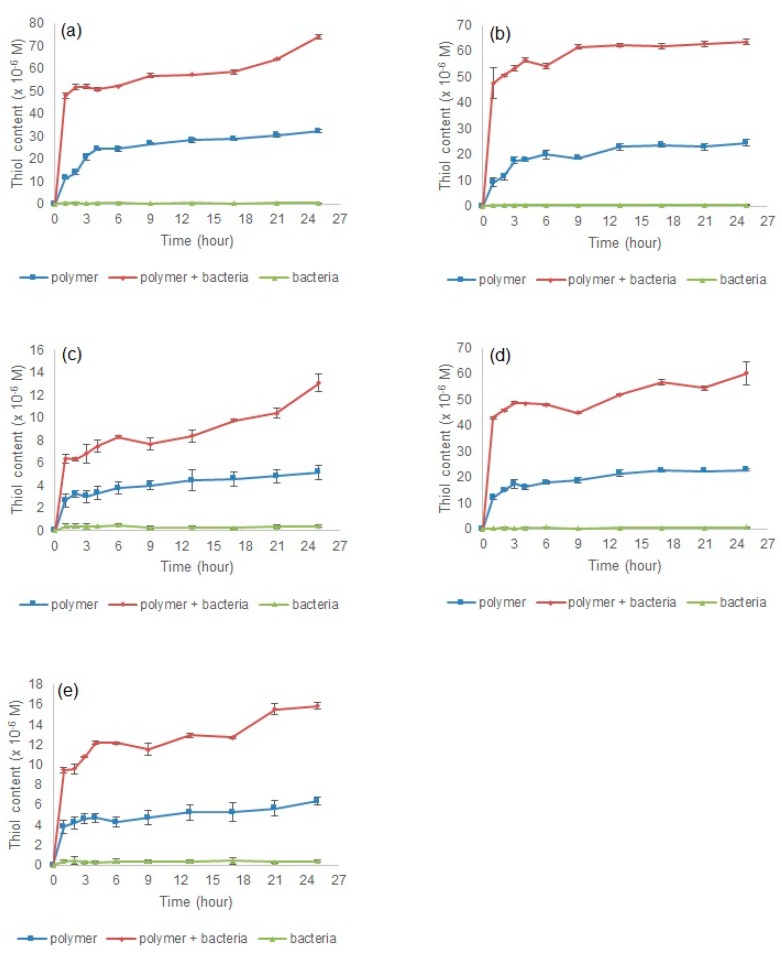
Thiol concentration in simulated colon condition for polymer (**a**) P10, (**b**) P15, (**c**) P21, (**d**) P25, and (**e**) P51.

Figure 7 shows the disintegration of disulphide cross-linked polymers in simulated stomach and intestine conditions. All polymers showed a gradual increase in thiol content until the fifth hour. Polymer P25 had the highest thiol content at 3.5 h with 37.491 × 10^−6^ M. This thiol content was contributed from the highest mole of disulphide bonds being reduced during P25 disintegration. Similar condition was observed from the previous study [18], where the highest thiol concentration was observed in disintegration from P15 polymer at 65 × 10^−5^ M compared to 15 × 10^−5^ M obtained from P11. The dissolution was then continued in the colon condition, where polymers from the stomach and intestine were placed in a visking tube containing *B. fragilis* to trigger the reducing environment of the colon. All polymers showed a similar pattern of disulphide disintegration (Figure 8). Higher thiol concentration was observed in polymers in the bacterial medium compared to polymers in the buffer medium only. These data showed that the disulphide polymers were reduced in media with low redox potential. There was no thiol detected from the bacteria only medium, which shows that no false results were obtained from polymer + bacteria medium. Polymers P10, P15, and P25 had similar thiol concentrations, and they were higher than those of P21 and P51. These results are related to the surface morphology of these polymers: P15 and P25 had a looser morphology compared to the tight and coarse surfaces of P21 and P51, whereas P10 had a large surface area that promoted reduction activity. The final thiol concentration and statistics results for each conditioned medium are summarised in Table 4 and Table 5.

## 4. Conclusions

In the present study, five different formulations of disulphide cross-linked polymers were successfully synthesised via the oxidative polymerisation method. All synthesised polymers were able to resist the harsh conditions of the upper GIT, but were then reduced to varying degrees in the simulated colon condition. Polymers P10 and P15 showed promising results for potential use as drug carriers for colon-targeted drug delivery. However, further investigations are needed to evaluate the safety of these polymers to deliver an effective treatment for various colon diseases.

## Figures and Tables

**Table 1:** Percentage of carbon, hydrogen, and nitrogen and melting point of synthesised compounds.

Compound	Carbon (%)		Hydrogen (%)		Nitrogen (%)		Melting point (°C)
	Theoretical	Actual	Theoretical	Actual	Theoretical	Actual	
**1**	78.90	78.74	6.58	7.00	4.39	4.35	94–96
**2**	77.63	76.53	5.66	5.96	3.77	3.69	225–227
**3**	46.51	45.66	5.43	5.60	10.85	9.73	159–161

**Table 2:** Physical appearance of synthesised disulphide polymers.

Polymers	Physical appearance
P10	White fine powder
P15	Yellowish film
P21	White coarse powder
P25	Sticky yellowish granules
P51	White solid

**Table 3:** Solubility of the synthesised polymers.

Solvent	Polymer				
	P10	P15	P21	P25	P51
DCM	Insoluble	Insoluble	Insoluble	Insoluble	Insoluble
DMSO	Soluble	Insoluble	Insoluble	Insoluble	Insoluble
Chloroform	Insoluble	Insoluble	Insoluble	Insoluble	Insoluble
Ethanol	Insoluble	Insoluble	Insoluble	Insoluble	Insoluble
Water	Insoluble	Insoluble	Insoluble	Insoluble	Insoluble

**Table 4:** Final thiol concentration (×10^−6^ M) of each simulated condition, mean ± SD, n = 3.

Incubation medium	P10	P15	P21	P25	P51
Stomach (a)	11.449 ± 0.967	11.402 ± 0.637	3.958 ± 0.872	19.458 ± 0.711	2.989 ± 0.743
Intestine (b)	15.533 ± 0.427	16.262 ± 0.737	6.485 ± 0.585	43.985 ± 0.556	7.048 ± 0.065
Colon (c)	74.108 ± 0.941	63.602 ± 1.004	13.056 ± 0.779	60.222 ± 4.279	15.861 ± 0.291
Statistical analysis	*p* < 0.05	*p* < 0.05	*p* < 0.05	*p* < 0.05	*p* < 0.05
Dunnett’s (significant)	a & c ^i^ b & c ^i^	a & c ^i^ b & c ^i^	a & c ^i^ b & c ^i^	a & c ^ii^ b & c ^ii^	a & c ^i^ b & c ^i^

^i^ = Dunnett’s t (2-sided) test, ^ii^ = Dunnett’s T3 test.

**Table 5:** Final thiol concentration (× 10^−6^ M) of different incubation of colon condition, mean ± SD, n = 3.

Incubation medium	P10	P15	P21	P25	P51
Bacteria (a)	0.297 ± 0.111	0.436 ± 0.194	0.363 ± 0.100	0.469 ± 0.247	0.385 ± 0.151
Polymer (b)	32.084 ± 0.818	24.551 ± 1.251	5.155 ± 0.632	22.748 ± 0.405	6.364 ± 0.369
Polymer + bacteria (c)	74.108 ± 0.941	63.602 ± 1.004	13.056 ± 0.779	60.222 ± 4.279	15.861 ± 0.291
Statistical analysis	*p* < 0.05	*p* < 0.05	*p* < 0.05	*p* < 0.05	*p* < 0.05
Dunnett’s (significant)	a & c ^i^ b & c ^i^	a & c ^i^ b & c ^i^	a & c ^i^ b & c ^i^	a & c ^ii^ b & c ^ii^	a & c ^i^ b & c ^i^

^i^ = Dunnett’s t (2-sided) test, ^ii^ = Dunnett’s T3 test.

## Supplementary materials

The following are available online at www.mdpi.com/2073-4360/9/8/311/s1.
