# Supplementary material for: Characterisation and Evaluation of Trimesic Acid Derivatives as Disulphide Cross-Linked Polymers for Potential Colon Targeted Drug Delivery
Source: Polymers (Basel). 2017 Jul 27;9(8):311. doi: 10.3390/polym9080311 (PMC6418558; doi:10.3390/polym9080311)
Supplement: Supplementary file 1 [file polymers-09-00311-s001.pdf]

Supplementary materials

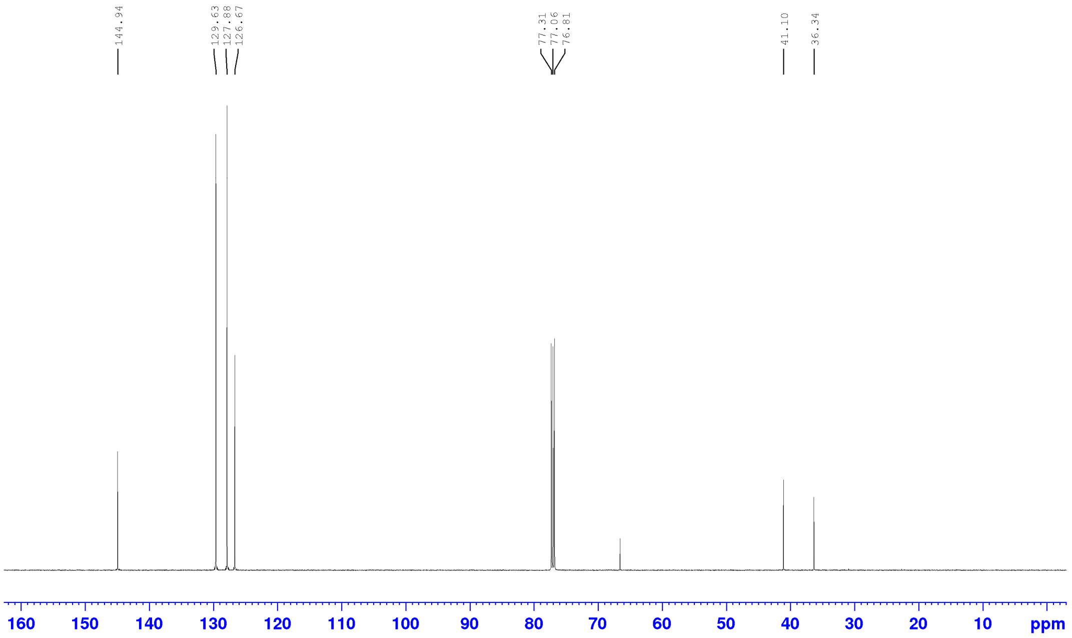

Figure S1: ^{13}C nuclear magnetic resonance (NMR) for compound 1

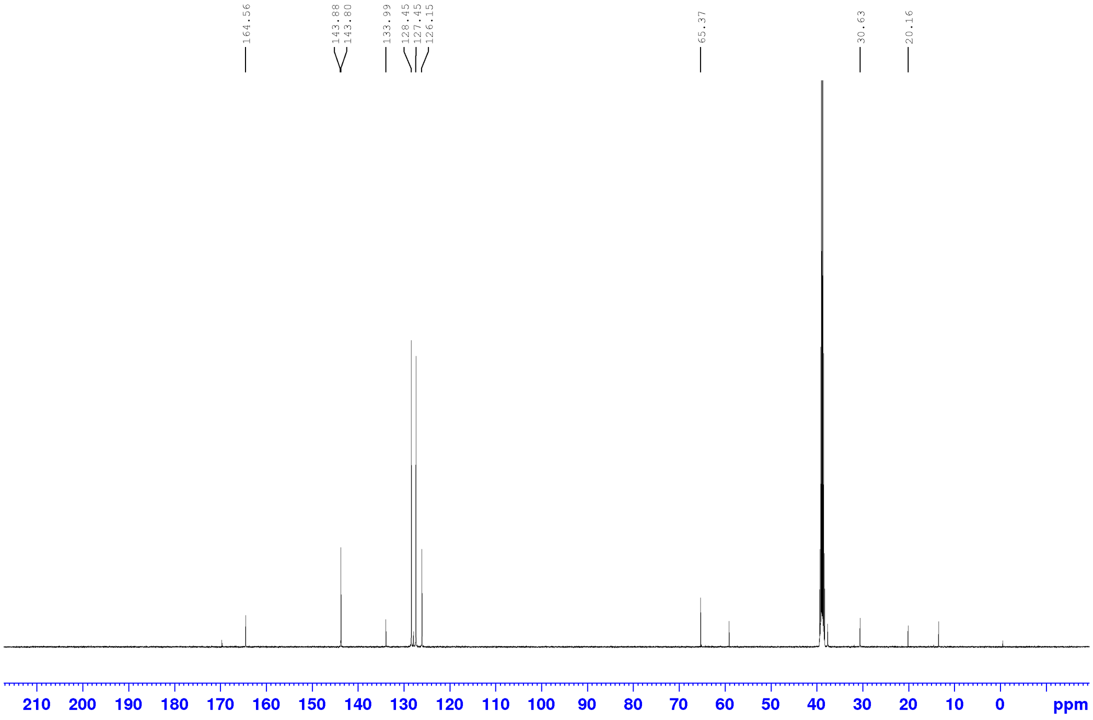

Figure S2: ^{13}C NMR for compound 2

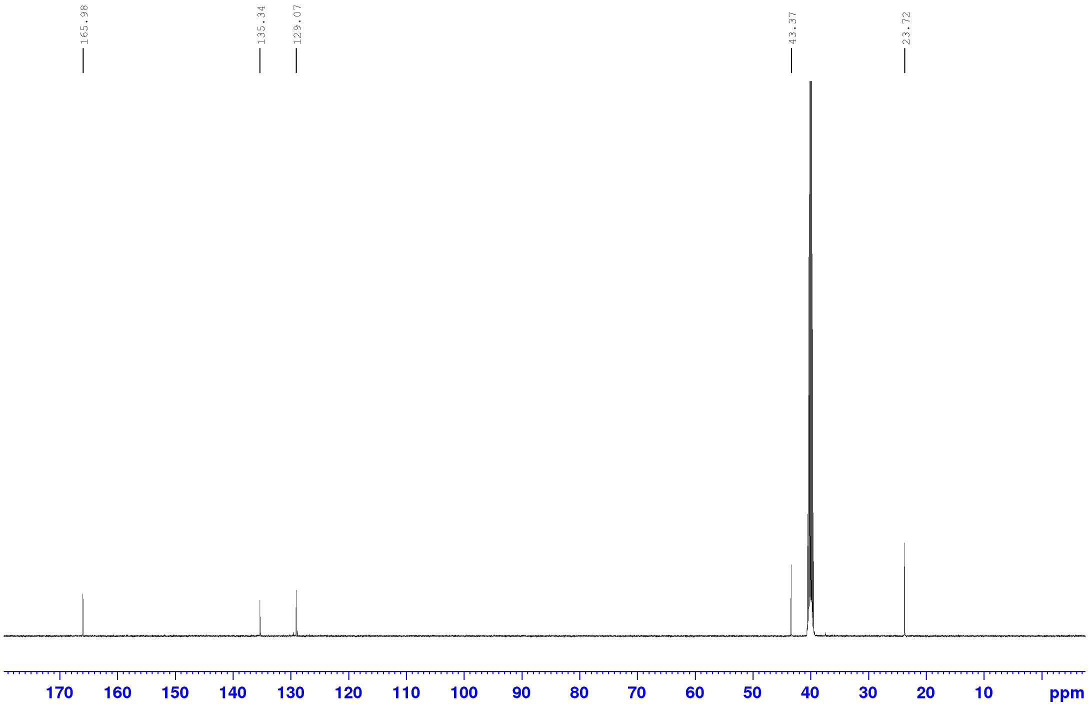

Figure S3: ^{13}C NMR for compound 3
